# The radial–tangential anisotropy of numerosity perception

**DOI:** 10.1167/jov.24.7.15

**Published:** 2024-07-24

**Authors:** Li L-Miao, Bert Reynvoet, Bilge Sayim

**Affiliations:** 1 Université de Lille, CNRS, UMR 9193–SCALab–Sciences Cognitives et Sciences Affectives, Lille, France; 2 Faculty of Psychology and Educational Sciences, KU Leuven Kulak, Kortrijk, Belgium; 3 Brain and Cognition, Faculty of Psychology and Educational Sciences, KU Leuven, Leuven, Belgium

**Keywords:** numerosity, spatial vision, crowding, redundancy masking, radial–tangential anisotropy, contrast polarity

## Abstract

Humans can estimate the number of visually presented items without counting. In most studies on numerosity perception, items are uniformly distributed across displays, with identical distributions in central and eccentric parts. However, the neural and perceptual representation of the human visual field differs between the fovea and the periphery. For example, in peripheral vision, there are strong asymmetries with regard to perceptual interferences between visual items. In particular, items arranged radially usually interfere more strongly with each other than items arranged tangentially (the radial–tangential anisotropy). This has been shown for crowding (the deleterious effect of clutter on target identification) and redundancy masking (the reduction of the number of perceived items in repeating patterns). In the present study, we tested how the radial–tangential anisotropy of peripheral vision impacts numerosity perception. In four experiments, we presented displays with varying numbers of discs that were predominantly arranged radially or tangentially, forming strong and weak interference conditions, respectively. Participants were asked to report the number of discs. We found that radial displays were reported as less numerous than tangential displays for all radial and tangential manipulations: weak (Experiment 1), strong (Experiment 2), and when using displays with mixed contrast polarity discs (Experiments 3 and 4). We propose that numerosity perception exhibits a significant radial–tangential anisotropy, resulting from local spatial interactions between items.

## Introduction

Humans are endowed with the ability to estimate the number of items in a visual scene without counting. It has been proposed that a dedicated system, the approximate number system, also known as the “number sense,” underlies such numerosity perception (Burr & Ross, 2008; Castaldi, Pomè, Cicchini, Burr, & Binda, 2021; Dehaene & Cohen, 1995; Dehaene, Dehaene-Lambertz, & Cohen, 1998; Feigenson, Dehaene, & Spelke, 2004). The approximate number system was proposed to be independent of other visual properties than the number of items (Burr & Ross, 2008). However, it was also suggested that visual properties (e.g., item size, density, convex hull, occupancy area) determine numerosity estimates (Allik, Tuulmets, & Vos, 1991; Aulet & Lourenco, 2021; Dakin, Tibber, Greenwood, Kingdom, & Morgan, 2011; Gilmore, Cragg, Hogan, & Inglis, 2016; Hurewitz, Gelman, & Schnitzer, 2006; Shilat, Salti, & Henik, 2021). Visual properties that are usually correlated with numerosity (Leibovich & Henik, 2013) are known factors modulating numerosity estimates. For example, varying the number of items necessarily goes hand in hand with changes of other visual properties, such as the occupancy area (Allik & Tuulmets, 1991), the convex hull (Gilmore et al., 2016; Katzin, Katzin, Rosén, Henik, & Salti, 2020; Katzin, 2018; Shilat et al., 2021), clustering (Bertamini, Guest, Vallortigara, Rugani, & Regolin, 2018; Bertamini, Zito, Scott-Samuel, & Hulleman, 2016), and density (Dakin et al., 2011; Durgin, 2008). All of these properties have been shown to play an important role in numerosity perception: Larger occupancy area, convex hull, and density were associated with larger numerosity estimates.

When multiple visual properties (such as convex hull and density) that contained information about the number of items were present, observers weighed these properties based on the perceptual integration of multiple visual cues, suggesting that multiple visual properties were integrated in numerosity perception (Gebuis & Reynvoet, 2012a; Gebuis & Reynvoet, 2012b). In particular, Gebuis and Reynvoet (2012a) demonstrated that observers use both informative and uninformative visual properties to estimate numerosity. In their study, displays were manipulated so that visual properties were either fully or partially correlated with numerosity. For example, in the partially congruent condition, larger numerosity displays had larger density but a smaller convex hull. In the fully congruent (incongruent) condition, all visual properties correlated positively (negatively) with numerosity; that is, visual properties were informative (uninformative) about the numerosity. Their study showed that performance was worse in the partially congruent condition, indicating that observers integrate multiple visual properties, when they are both informative or uninformative.

Studies investigating numerosity perception usually use displays that consist of multiple items that cover a relatively large area of the visual field, including the fovea, parafovea, and periphery (e.g., Anobile, Turi, Cicchini, & Burr, 2015; Chakravarthi & Bertamini, 2020; Mengal & Matathia, 1980; Valsecchi, Toscani, & Gegenfurtner, 2013). However, there are important differences among foveal, parafoveal, and peripheral vision (Knotts, Odegaard, Lau, & Rosenthal, 2019; Larson & Loschky, 2009; Rosenholtz, 2016; Strasburger, Rentschler, & Jüttner, 2011). For example, crowding, the interference of neighboring objects on target perception occurs over much larger distances in the periphery than in the fovea (Andriessen & Bouma, 1976; Bouma, 1970; He, Cavanagh, & Intriligator, 1996; Herzog, Sayim, Chicherov, & Manassi, 2015; Levi, 2008; Levi, Klein, & Aitsebaomo, 1985; Pelli, Palomares, & Majaj, 2004; Pelli & Tillman, 2008; Sayim, Greenwood, & Cavanagh, 2014; Sayim & Wagemans, 2017; Sayim, Westheimer, & Herzog, 2008; Sayim, Westheimer, & Herzog, 2010; Strasburger et al., 2011; Whitney & Levi, 2011). Recently, it was suggested that crowding plays a role in numerosity perception (Anobile, Burr, Gasperini, & Cicchini, 2015; Valsecchi et al., 2013; see also L-Miao, Reynvoet, & Sayim, 2022). For example, Valsecchi et al. (2013) presented two adjacent dot clouds and asked participants to fixate on the center of one of the clouds (hence, the other dot cloud was located in the periphery). Participants indicated which of the dot clouds contained more dots. Their results showed that the perceived numerosity of the peripheral dot clouds was lower compared with the foveal dot clouds. The magnitude of the foveal–peripheral difference increased with increasing eccentricity, suggesting that eccentricity determined underestimation of the peripheral dot clouds. Based on these results, Valsecchi et al. (2013) suggested that crowding underlies numerosity underestimation in peripherally presented displays (see also Anobile et al., 2015).

Although the typical underestimation in numerosity studies suggests a detection-like error—some items are not detected and are missing from the estimates—crowding is usually assumed to interfere with target identification but not target detection (Levi, Hariharan, & Klein, 2002; Pelli et al., 2004). However, crowding could still underlie underestimation if crowded items were perceptually merged with (i.e., not segmented from) neighboring items. An alternative explanation for underestimation in peripheral vision is redundancy masking (RM), a recently discovered phenomenon in which the number of perceived items in repeating patterns is lower than the number of presented items (Sayim & Taylor, 2019; Sayim et al., 2022; Yildirim, Coates, & Sayim, 2020; Yildirim, Coates, & Sayim, 2021; Yildirim, Coates, & Sayim, 2022; Yildirim-Keles, Coates, & Sayim, 2024). For example, when presenting an array of identical, radially arranged lines in the visual periphery, observers often report fewer lines than were presented, even with as few as three lines (Yildirim et al., 2020; Yildirim et al., 2021). Hence, RM, in contrast to crowding, is characterized by detection-like errors (for diminishment or detection-like errors in crowding paradigms, see also Coates, Wagemans, & Sayim, 2017; Sayim & Wagemans, 2017). RM has recently been suggested to modulate numerosity perception (L-Miao et al., 2022).

**Figure 1.**
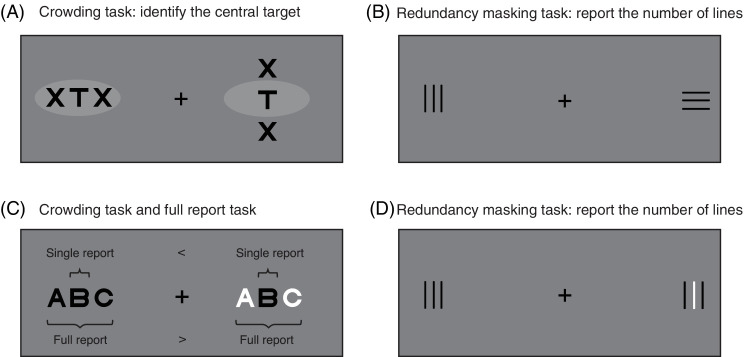
Illustration of the radial–tangential anisotropy of crowding (**A**) and redundancy masking (**B**) and the effect of same versus opposite contrast polarity in crowding (**C**) and redundancy masking (**D**). (**A**) When fixating the fixation cross, the identification of a target (T; left) in the visual periphery is usually strongly impaired by flankers that are radially positioned in the interference zone (indicated by the shaded ellipse). Flankers cease to interfere with target perception at smaller target–flanker spacings when placed tangentially (right). (**B**) When a line triplet is arranged radially (left), most observers report a line pair. When the line triplet is arranged tangentially (right), observers usually do not report lower numbers of lines (Yildirim et al., 2020). (**C**) Target identification is often strongly impaired by flankers of the same contrast polarity (left) compared with flankers of opposite contrast polarity (right) when a single target (**B**) needs to be reported. However, superior performance has been shown for uniform compared with opposite contrast polarity when reporting all items (full report) (Rummens & Sayim, 2021). (**D**) When three radially arranged items are presented, the reported number of items is usually lower when all items have the same (left) compared with opposite (right) contrast polarity (Hansmann-Roth & Sayim, 2023).

Both crowding and RM are subject to a strong radial–tangential anisotropy (Feng, Jiang, & He, 2007; Greenwood, Szinte, Sayim, & Cavanagh, 2017; Kwon, Bao, Millin, & Tjan, 2014; Pelli & Tillman, 2008; Petrov & Meleshkevich, 2011; Toet & Levi, 1992; Yildirim et al., 2020; Yildirim et al., 2022). In crowding, radially placed flankers interfere more strongly with target perception than tangentially placed flankers (Figure 1A). Similarly, RM has been shown for radially arranged but not tangentially arranged items (Figure 1B) (Yildirim et al., 2022). In a previous study, testing several large numerosities (from 21 to 58), we used displays with predominantly radially or tangentially arranged items and found that numerosity estimation was similarly subject to a radial–tangential anisotropy: Estimates were lower when items were arranged radially compared with tangentially (L-Miao et al., 2022). In the present study, we systematically varied the extent of radial versus tangential arrangements to further investigate the role of local spatial interactions between items and the radial–tangential anisotropy in numerosity perception.

Furthermore, again building on previous research in peripheral vision, we varied the similarity of the displayed items. Both crowding and RM have been shown to depend on item similarity, with higher similarity usually yielding stronger crowding (Chakravarthi & Cavanagh, 2007; Chung, Legge, & Tjan, 2002; Chung, Levi, & Legge, 2001; Chung & Mansfield, 2009; Kooi, Toet, Tripathy, & Levi, 1994) and stronger RM (Hansmann-Roth & Sayim, 2023; Sayim et al., 2022). For example, crowding is typically stronger when the target and the flankers are of the same contrast polarity (i.e., all items either black or white) compared with when they have opposite contrast polarity (target black and flankers white or vice versa) (Figure 1C, single report) (Chakravarthi & Cavanagh, 2007; Chung & Mansfield, 2009; Sayim et al., 2008; but see Rummens & Sayim, 2019; Rummens & Sayim, 2021; Rummens & Sayim, 2022). Importantly, this “polarity advantage” has mainly been observed in experiments where a single target flanked by two or more flankers had to be identified (or discriminated); that is, not all items of the stimuli were task relevant. Recent studies, in which all (three) parts of a stimulus were task relevant, have revealed either the absence of a polarity advantage (Figure 1C, full report) (Rummens & Sayim, 2021; Rummens & Sayim, 2022) or a disadvantage when identifying stimuli with opposite compared with uniform contrast polarity (Rummens & Sayim, 2019). In most numerosity studies where participants report the number of items, all items are task relevant. Hence, estimating the number of items of opposite contrast polarity would not readily be expected to yield more accurate estimations if crowding was the cause of underestimation in numerosity perception. In RM paradigms, by contrast, all items are task relevant, as in typical numerosity studies, and opposite contrast polarity of neighboring lines (in particular, in line triplets) has been shown to reduce the magnitude of RM compared with uniform triplets (Figure 1D) (Hansmann-Roth & Sayim, 2023). Hence, RM predicts more accurate numerosity estimation with opposite compared with uniform contrast polarity.

Numerosity studies investigating the impact of contrast polarity on numerosity estimation have yielded inconsistent results. Whereas some studies have shown that varying contrast polarity had no impact on numerosity perception, others have shown a strong impact. For example, Tibber, Greenwood, & Dakin (2012) found similar numerosity estimates for uniform and mixed contrast polarity displays (see also Dakin et al., 2011). In contrast, Chakravarthi and Bertamini (2020) found that contrast polarity modulated numerosity perception, but only in low-density and not high-density displays. Taken together, these results show that mixed contrast polarity may (a) improve performance in crowding paradigms (possibly only when identifying a single target among irrelevant flankers), (b) deteriorate performance in crowding paradigms (when all items are task relevant), (c) reduce RM, and (d) have varying influences on numerosity perception.

The primary aim of the experiments reported here is to investigate the impact of the radial–tangential anisotropy in peripheral vision on numerosity perception. Specifically, we sought to determine how the arrangement of items (radial vs. tangential) affects numerosity estimation. Our experiments build on previous findings in crowding and RM, which have demonstrated strong radial–tangential anisotropies. We systematically varied the extent to which items were arranged radially and tangentially, including displays to maximize and minimize the probability of local interactions between items: Displays with predominantly radially arranged items were expected to yield lower numerosity estimates than displays with predominantly tangentially arranged items.

Furthermore, we varied the contrast polarity of neighboring items to investigate whether there was an interaction between the two factors of arrangement (radial vs. tangential) and contrast polarity (uniform vs. mixed). Specifically, we hypothesized that radial arrangements would (and tangential arrangements would not) be affected by mixed compared with uniform contrast polarity if RM underlied underestimation in numerosity perception. In radial arrangements, interference between neighboring items that yields strong RM with uniform contrast polarity items is expected to yield much weaker RM with mixed contrast polarity items. In contrast, there is no RM with uniform contrast polarity items in tangential arrangements (Yildirim et al., 2020); therefore, mixed contrast polarity is not expected to reduce RM in tangential arrangements. Hence, we expected a strong difference between radial and tangential arrangements with uniform contrast polarity items and a smaller (or no) difference with mixed contrast polarity items. Alternatively, similar results with uniform and mixed contrast polarity displays (no interaction between arrangement and contrast polarity) would be expected if the reduction of interference by mixed contrast polarity was similarly strong in radial and tangential displays or if there was no reduction.

Finally, grouping has been suggested to impact numerosity perception. Items connected by, for example, lines are usually judged as less numerous compared with non-connected items (Franconeri, Bemis, & Alvarez, 2009; Kirjakovski & Matsumoto, 2016). In addition to modulating local spatial interactions, our contrast polarity manipulation was also used to explore the effects of the global grouping structure. In particular, the global grouping structure that occurs when arranging items predominantly radially (concentric patterns) or tangentially (ray patterns) may be reduced with mixed contrast polarity items. Hence, if grouping determined differences between radial and tangential arrangements, this difference would be expected to be reduced (or disappear) with mixed contrast polarity.

## Experiment 1. Weak radial and tangential arrangements

In Experiment 1 (online), we tested how the radial–tangential anisotropy modulated numerosity estimation by presenting displays that were arranged predominantly radially or tangentially. We refer to the manipulation in Experiment 1 as “weak” radial and tangential arrangements and the manipulation in Experiment 2 as “strong” radial and tangential arrangements (see below). With different groups of participants, small numerosities (34–44) and large numerosities (54–64) were tested in Experiment 1a and Experiment 1b, respectively. Note that the terms “small” and “large” numerosities refer to the relative number of items within the same experiment.

### Method

#### Participants

The number of participants in all experiments was determined by a simulation-based power analysis (Kumle, Võ, & Draschkow, 2021), using the mixedpower package in R (R Foundation for Statistical Computing, Vienna, Austria). We ran 1000 repetitions in the final simulation for each experiment. Participants were recruited online using Prolific (www.prolific.co) for Experiment 1 and Experiment 3. Forty participants were recruited for Experiment 1a. We removed six participants: four participants did not complete the study, one participant had more than 5% of invalid responses (e.g., meaningless numbers such as ‘000’), and one participant failed the attentional check (performance in subitizing trials was lower than 90% correct; details below). Data from 34 participants (16 males and 18 females; mean age, 25.3 years; range, 18–38 years) were submitted for analysis in Experiment 1a. Forty participants who did not participate in Experiment 1a were recruited for Experiment 1b. We removed eight participants: two participants did not complete the study and the other six participants failed the subitizing attention check. Data from 32 participants (11 males and 21 females; mean age, 25.7 years; range, 19–39 years) were submitted for analysis in Experiment 1b. The statistical power of our fixed factor (arrangement) exceeded 0.98 in both experiments. All participants were naïve as to the purpose of the study and received monetary compensation (7.5 £/hr). All participants reported normal or corrected-to-normal visual acuity. Informed consent was obtained from participants prior to the experiment. All experiments were approved by the ethics committee of the Ulille SHS, University of Lille.

#### Apparatus and stimuli

The experiments were created using PsychoPy coder v3.1.2 (Peirce, 2009). The online experiments (Experiments 1a, 1b, 3a, 3b) were hosted by Pavlovia (www.pavlovia.org), and participants were instructed to use a 24-inch monitor with a vertical resolution of 1080 pixels viewed from a distance of about 45 cm (1 pixel corresponded to 0.04°). The analysis codes and data are available at https://github.com/miaoli-psy/ms2_numerosity.

**Figure 2.**
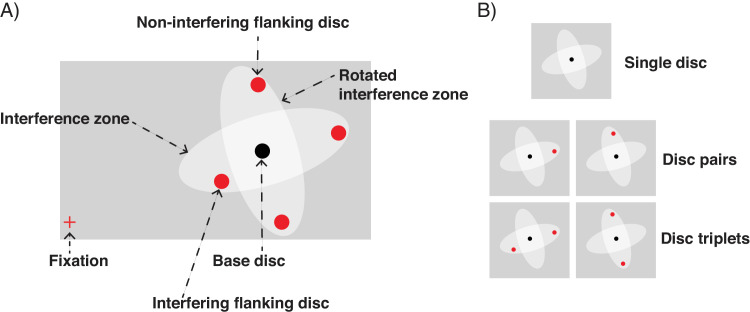
(**A**) Illustration of the display construction. In radial displays, flanking discs were added to the interference zone of the base discs. In tangential displays, flanking discs were added to the rotated interference zone of the base discs. (**B**) Possible disc configurations.

Stimuli consisted of black (Hex Code #000000) discs (radius 0.36°) presented on a gray (Hex Code #B6B6B6) background. All discs were presented within an imaginary rectangular region that occupied 40% (Experiment 1a) or 60% (Experiment 1b) of the screen. No disc was presented within a circular region (radius 4°) around fixation. For stimulus creation, discs were either base discs or flanking discs (Figure 2A). To create predominantly radially or tangentially arranged displays, each base disc was surrounded by a radially oriented interference zone and a concentric 90° rotated (i.e., tangentially oriented) interference zone of the same size (these zones were only used to construct the displays and were never shown to participants) (Figure 2A). The major and the minor axes of the interference zones were 0.25× eccentricity and 0.1× eccentricity, respectively. The size of the zones was determined based on common estimates of the size of the interference zone in crowding (Toet & Levi, 1992) and was used to determine the distance among discs in the displays. The two interference zones were free from other base discs. The flanking discs were placed into either the radially or tangentially oriented zones to form radial or tangential displays, respectively. No flanking disc was added to the overlap area of the two interference zones. In Experiment 1, each base disc was presented without any flanking disc (remaining a single disc), paired with one flanking disc (forming a disc pair), or paired with two flanking discs (forming a disc triplet) (Figure 2B). We varied the percentage of single discs, disc pairs, and disc triplets to reduce the probability that participants estimated the number of discs by multiplication of the number of estimated disc pairs and/or triplets. The percentage of disc pairs varied between 0% and 100% in steps of 25% (resulting in five conditions). In each condition, 0%, 25%, 50%, 75%, or 100% of the base discs (randomly selected) were paired with one flanking disc (forming disc pairs). The base discs that were not paired were equally divided between two configurations; half of these discs were presented with two flanking discs (forming disc triplets), and the other half were presented without any flanking discs (remaining single base discs). For example, if the display had 50% disc pairs, 50% of the base discs were paired with one flanking disc. The remaining 50% of base discs were split equally, with 25% forming disc triplets and 25% single base discs.

**Figure 3.**
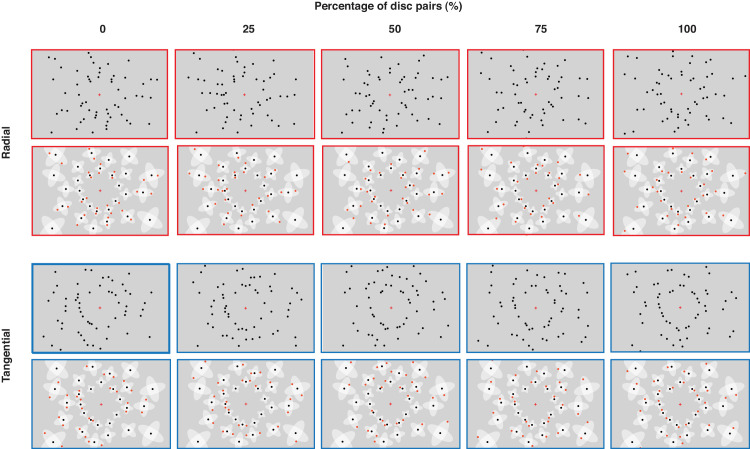
Illustration of displays in the radial and tangential conditions (first and third rows, respectively) and their geometric principles (second and last rows, respectively) in Experiment 1. Note that all displays in the figure share the same base discs for illustration purposes (in the experiments, no display shared the same base discs, as each display was generated independently). The red and blue frames of the displays correspond to radial and tangential arrangements, respectively (not presented in the experiments). Illustrations in Figures 5, 7, and 9 follow the same principles.

Figure 3 illustrates the displays used in Experiment 1. To generate a display, a random position was selected to place the first base disc with its two corresponding interference zones (Figure 2A). Additional base discs were added iteratively on the displays with the constraint that no interference zones of the base discs overlapped. Base discs were positioned on the display until no disc without overlapping (rotated) interference zones could be added anymore. Flanking discs were added into the interference zones or rotated interference zones (excluding the central, overlapping zone) to form radial and tangential displays, respectively. All discs on the displays were presented within a rectangular region. The size of the rectangular region was either small (21.5° width × 13.5° height occupying 40% of the entire screen; Experiments 1a, 2a, 3a, and 4: small numerosities) or large (27.0° width × 18.5° height occupying 60% of the entire screen; Experiments 1b, 2b, 3b, and 4: large numerosities). The size of the rectangular region determined the maximum number of base discs that could be presented, and, therefore, determined the tested numerosities. For each percent of disc pairs condition, we generated 10,000 displays (5000 radial and 5000 tangential displays). We selected displays with the same numerosity and ensured that radial and tangential displays matched with regard to average eccentricity, average spacing, convex hull, occupancy area, and density (see Supplementary Table S1). There were two tested numerosity ranges. The small numerosity range contained 34 to 44 discs (with increments of two), including 17 to 22 base discs (Experiments 1a, 3a, and 4). The large numerosity range contained 54 to 64 discs (with increments of two) including 27 to 32 base discs (Experiments 1b, 3b, and 4).

#### Design and procedure

The design and procedure of Experiment 1a and Experiment 1b were identical. Each trial started with a red fixation cross (0.2° × 0.2°) presented at the center of the screen. Observers initiated each trial by pressing the spacebar. Each display was presented for 150 ms. Participants were required to enter their best estimates of the number of discs using the number keys on the keyboard. The estimates entered by participants were displayed on the screen for each trial. There was no feedback, and there was no time limit for participants to respond. Prior to the experiment, participants viewed five reference displays. The numerosities of the five reference displays were equally distributed around the mean numerosity of each experimental block (±0.125 and ±0.25 times the mean numerosity of the block). For example, if the mean numerosity of the block was 40, then the five selected reference displays had numerosities of 30, 35, 40, 45, and 50, respectively. Each reference display was presented for 150 ms, and participants were informed about the actual number of the reference display after the offset of the reference display. Participants were not informed about the numerosity ranges for the experiment. Each participant performed 300 trials (50 trials for each numerosity in random order). The experiment was interspersed with 30 trials with numerosities in the subitizing range (2–4 discs), where individuals can typically respond quickly and accurately (Atkinson, Campbell, & Francis, 1976; Kaufman, Lord, Reese, & Volkmann, 1949). These trials were incorporated for attentional control. Participants who responded incorrectly on 10% or more of the control trials were excluded from the experiment.

#### Data analysis

We calculated the deviation score (DV) by subtracting the actual numerosity of the display from the reported numerosity for each trial. We used the DV with its sign in all analyses with individual analysis models for each experiment. This approach enabled us to discern over- and underestimations. When a comparison of the accuracy of numerosity estimates was required, we use the absolute value of the DV. The raw data were cleaned and organized using R 3.6.3 and RStudio. Linear mixed-effect analyses were conducted using the lme4 package (Bates, Mächler, Bolker, & Walker, 2015). The emmeans package (Lenth, Singmann, Love, Buerkner, & Herve, 2021) was used for estimation statistics and post hoc comparisons on the full model.

To examine differences in the DV between the radial and tangential arrangements in Experiment 1a, we included arrangement (radial vs. tangential) as a fixed factor. We used models with both random slope and random intercept, assuming that the effect of the arrangement can vary between participants and tested numerosities. To compare the radial with the tangential arrangements, simple contrast coding was used, setting the radial arrangement to –0.5 and the tangential arrangement to 0.5. The estimates obtained from our model using simple contrast coding directly reflect the difference between the radial and tangential arrangement. The analysis for Experiment 1b was identical to that for Experiment 1a.

### Results

#### Experiment 1a: Small numerosities (34–44)

**Figure 4.**
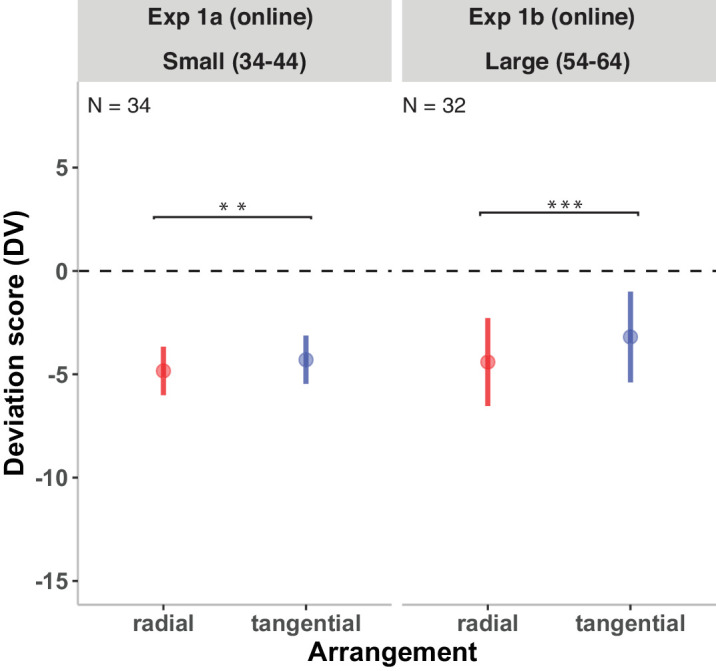
Results of Experiment 1a (left panel) and Experiment 1b (right panel). DVs of the radial and tangential conditions are shown. DVs of 0 represent correct estimates; negative DVs, underestimations; and positive DVs, overestimation. Error bars indicate ±1 *SEM*. Significant fixed effects are denoted by **p < 0.01 and ***p < 0.001.

Figure 4 (left panel) shows the DVs of the radial and tangential arrangements in Experiment 1a. The model revealed a significant effect of arrangement on DVs, *b* = 0.89 ± 0.25, *t*(33.00) = 3.47, *p* < 0.005, with lower reported numerosities in radial compared with tangential displays. Because we used simple contrast coding for arrangement, the observed coefficient (*b* = 0.89) indicates that, holding other factors constant, the difference in DVs between the radial and tangential arrangements was 0.89.

#### Experiment 1b: Large numerosities (54–64)

Figure 4 (right panel) shows the DVs of the radial and tangential arrangements in Experiment 1b. The results revealed a significant effect of arrangement on DVs, *b* = 1.35 ± 0.31, *t*(31.00) = 4.32, *p* < 0.001, with lower reported numerosities in radial compared with tangential displays (*b* = –1.35; confidence interval [CI], –2.01 to –0.70).

## Experiment 2. Strong radial and tangential arrangements

**Figure 5.**
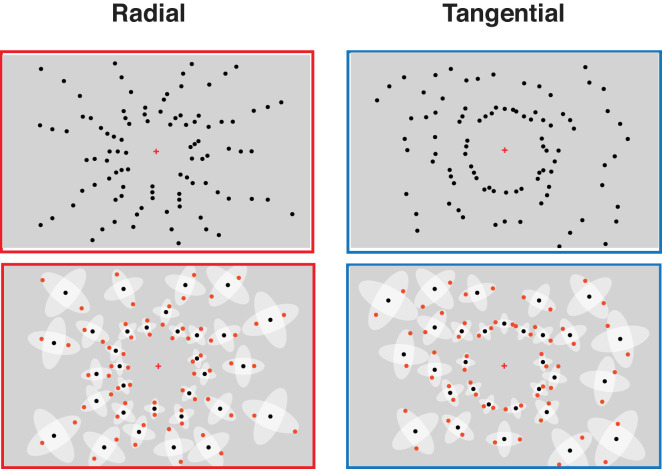
Illustration of displays in the radial and tangential arrangements (upper left and upper right, respectively) and their geometric principles (lower left and lower right, respectively) in Experiment 2.

In Experiment 2 (in-lab), we created displays to maximize the probability of interference among discs in the radial arrangement. Unlike in Experiment 1 (weak radial and tangential arrangements) where, on average, one flanking disc was placed in an interference zone of a base disc, in Experiment 2 two flanking discs were placed in the interference zone (or rotated interference zone) of each base disc, forming disc triplets (Figure 5).

### Method

#### Participants

The simulation power analysis showed that the power of the fixed factor arrangement reached 0.99 with 15 participants. We recruited 16 participants (13 females and three males; mean age, 20.4 years; range, 19–23 years). All participants were undergraduate psychology students at KU Leuven. They received course credits for their participation. Performance of all participants was more than 95% correct in the subitizing trials. No participant was removed from the analysis.

#### Apparatus and stimuli

The experiment was created using PsychoPy 3.1.2 (Peirce, 2009) and run on a desktop PC. All stimuli were presented on an light-emitting diode 24-inch display, with a resolution of 1920 × 1080 pixels and a refresh rate of 60 Hz. During the experiment, participants sat in front of the screen at approximately 45 cm (1 pixel corresponded to 0.04°). The experiments were conducted in a dimly lit experiment room.

Figure 5 illustrates the displays used in Experiment 2. As in Experiment 1, the base discs were surrounded by the two interference zones. To maximize the probability of interference among discs, two flanking discs (instead of one) were added into the interference zone, forming “disc triplets” (see Figure 2B). Flanking discs were added into either the interference zone or the rotated interference zone (never in both), yielding radial and tangential displays. There were two display sizes (small and large). The numerosities were 51, 54, 57, 60, 63, 69, and 72 in the small displays, and 78, 81, 84, 87, 90, 93, 96, and 99 in the large displays.

#### Design and procedure

The design and procedure were identical to those for Experiment 1 except that each participant was presented with both small (51–72) and large (87–99) numerosity displays. Each participant performed six blocks in random order (three large numerosities and three small numerosities) of 80 trials.

#### Data analysis

The data analysis was identical to that for Experiment 1, except for the following changes. Arrangement (radial vs. tangential) and numerosity (small vs. large) were submitted as fixed factors in the model. We examined the interaction effect between arrangement and numerosity on DVs by performing likelihood ratio tests between models with and without the interaction term (Winter, 2013). In the case of a significant interaction effect, the DV differences under each numerosity (small or large) with a contrast comparison would be examined. In the case of a non-significant interaction effect, the interaction factor would be removed from the full model, examining the effect of arrangement and numerosity on DVs separately. We applied a simple contrast coding scheme for arrangement and numerosity: Radial arrangement and small numerosity were assigned a contrast value of –0.5, and tangential arrangement and large numerosity were assigned a contrast value of 0.5.

We also explored whether there were any differences between Experiment 1 and Experiment 2 using a linear mixed-model analysis, with arrangement (radial vs. tangential) and experiment (Experiment 1 vs. Experiment 2) as fixed factors and random intercepts for arrangement that accounted for possible baseline differences across participants in radial and tangential displays. For between-experiments comparisons, we built two models. One model used the DV as the model outcome, and the other used the absolute value of DV as the model outcome. In the model construction, the outcome of the former model was the DV, yielding estimates with signs. The outcome of the latter model was the absolute value of the DV, allowing us to compare the accuracy of the estimates. In the latter model, a smaller value indicates greater accuracy.

### Results

#### Experiment 2

**Figure 6.**
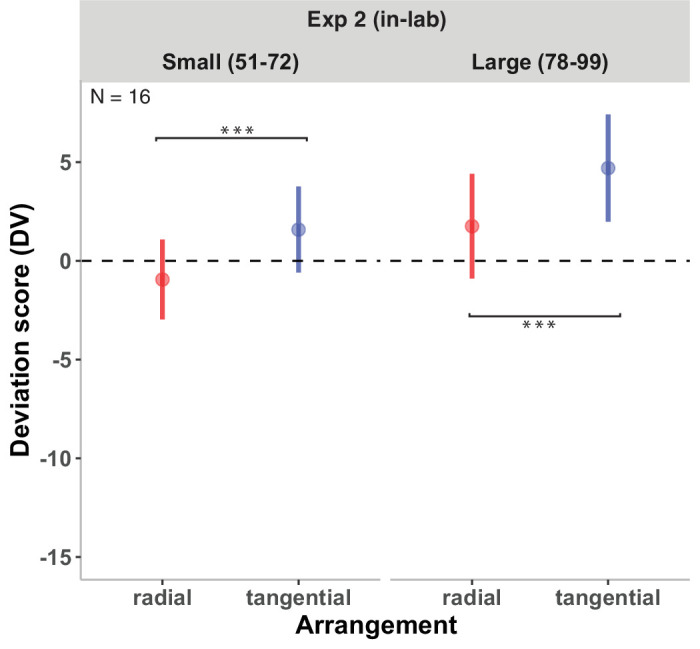
Results of Experiment 2. DVs as a function of numerosity are shown. DVs of 0 represent correct responses; negative DVs, underestimation; and positive DVs, overestimation. Error bars indicate ±1 *SEM*. Significant fixed effects are denoted by ***p < 0.001.

Figure 6 shows the DVs of the radial and tangential arrangements of Experiment 2. The model comparison between the full model (including the interaction between arrangement and numerosity as a fixed factor) and the reduced model (excluding the interaction as a fixed factor) revealed no difference, χ^2^(1) = 0.25, *p* = 0.62, indicating no significant interaction between arrangement and numerosity. The fixed-effect analysis revealed a significant influence of arrangement on DVs, *b* = 2.73 ± 0.85, *t*(15) = 3.24, *p* < 0.01, showing that the DVs of radial displays were lower compared with tangential displays. No effect of numerosity on DVs was found, *b* = 2.91 ± 2.14, *t*(15) = 1.36, *p* = 0.20.

#### Comparison of Experiment 1 and Experiment 2

The comparison between Experiment 1 and Experiment 2 revealed a trend for lower deviation scores in Experiment 1 (*b* = –3.97; CI, –6.26 to –1.68) than in Experiment 2 (*b* = 0.80; CI, –3.78 to 5.37), *t*(80.57) = 1.83, *p* = 0.07. For the accuracy of the estimates, the model that used the absolute value of the DV as the dependent variable, revealed that the main effect of experiment was not significant, *b* = 1.97 ± 1.38, *t*(80.5) = 1.42, *p* = 0.16. The effect of arrangement (lower estimates for tangential than radial arrangements) was also present when combining the data of Experiment 1 and Experiment 2, *b* = 1.25 ± 0.25, *t*(84.7) = 4.92, *p* < 0.001.

## Experiment 3. Radial and tangential arrangements with mixed contrast polarity

In Experiment 3 (online), we investigated whether differences between the radial and the tangential arrangements were impacted when using mixed contrast polarity displays.

### Method

#### Participants

Forty participants who were naïve to the aims of the study were recruited for Experiment 3a. We removed 11 participants: 10 did not complete the study and one failed the subitizing attention check (performance in subitizing trials was lower than 90% correct). Data from 29 participants (20 females, eight males, and one participant who did not indicate any sex; mean age, 25.4 years; range, 19–38 years) were submitted for analysis in Experiment 3a. For Experiment 3b, 40 naïve participants were recruited. We removed 12 participants: two did not complete the study and 10 failed the subitizing attention check. Data from 28 participants (19 females and nine males; mean age, 27.3 years; range, 19–40 years) were submitted for analysis in Experiment 3b. The statistical power of our fixed factors (arrangement and contrast polarity) exceeded 0.98 in both experiments.

#### Apparatus and stimuli

**Figure 7.**
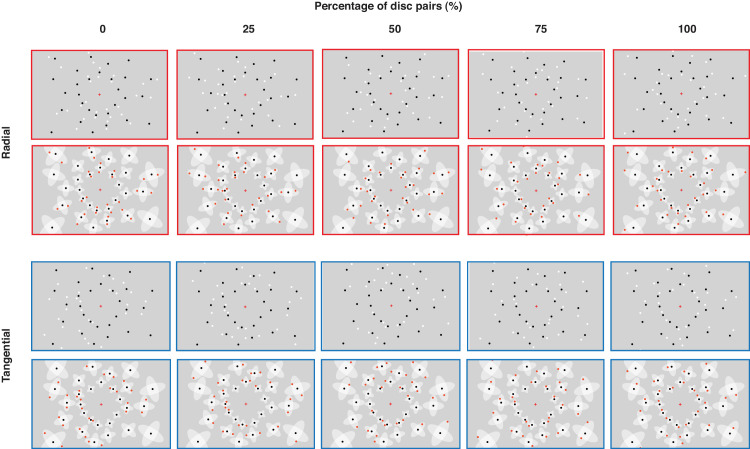
Illustration of displays in the radial and tangential conditions (first and third rows, respectively) and their geometric principles (second and last rows, respectively) in Experiment 3.

The apparatus was identical to that for Experiment 1a. The stimuli used in Experiments 3a and 3b were identical to those for Experiments 1a and 1b, respectively, except that the displays were of mixed contrast polarity (black and white discs; all base discs were black, all flanking discs were white) (Figure 7).

#### Design and procedure

The design and procedure were identical to those for Experiment 1.

#### Data analysis

The data analyses for Experiments 3a and 3b were the same as in Experiments 1a and 1b, respectively. The only difference is a simpler random effect term that allowed the baseline of DVs and the effect of the arrangement to vary by participant. Additionally, we compared the results of Experiments 1 and 3 (Experiment 2 was excluded as there was a higher number of displays than in Experiments 1 and 3). The comparison model used in this analysis was the same as the one used for comparing Experiments 1 and Experiment 2, with the exception that experiment was coded using sum contrast to compare against the grand mean.

### Results

#### Experiment 3a: Small numerosities (34–44)

**Figure 8.**
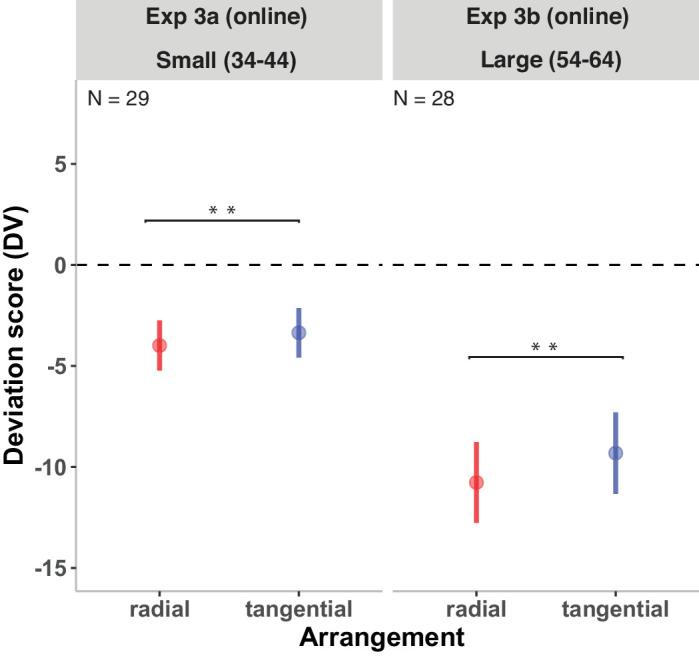
Results of Experiment 3a (left panel) and Experiment 3b (right panel). DVs of the radial and tangential arrangements are shown. DVs of 0 represent correct estimates; negative DVs, underestimations; and positive DVs, overestimations. Error bars indicate ±1 *SEM*. Significant fixed effects are denoted by **p < 0.01 and ***p < 0.001.

Figure 8 (left panel) shows the DVs for the radial and tangential arrangements in Experiment 3a. The effect of arrangement on DVs was significant, *b* = 0.65 ± 0.25, *t*(31.00) = 3.67, *p* < 0.001. This result indicates that, when other factors were held constant, the DV difference between radial and tangential arrangements was 0.65.

#### Experiment 3b: Large numerosities (54–64)

Figure 8 (right panel) shows the DVs of the radial and tangential arrangements in Experiment 3b. The DVs of radial displays were significantly lower compared with the DVs of tangential displays, *b* = 1.44 ± 0.41, *t*(28) = 3.57, *p* < 0.005.

#### Comparison of Experiment 1 and Experiment 3

We observed that the grand mean deviation score (i.e., the mean DV across 4 experiments: Experiments 1a, 1b, 3a, and 3b) was significantly below zero (*b* = –5.99 ± 0.84, *p* < 0.001). The overall negative estimate indicates that, on average, there was a tendency for participants to underestimate the number of items across the four experiments. The DVs of Experiment 1b were significantly higher than the grand mean, *b* = 2.70 ± 0.90, *t*(158.68) = 3.00, *p* < 0.01, showing that higher estimates (closer to 0) in Experiment 1b compared with the average of all experiments.

## Experiment 4: Radial and tangential arrangements with uniform and mixed contrast polarity

In Experiment 4 (in-lab), we compared the uniform and mixed contrast polarity displays with a within-subjects design. Participants were presented with both uniform and mixed contrast polarity displays.

### Method

#### Participants

The simulation power analysis showed that the power of the fixed factors (arrangement and contrast polarity) reached 0.99 with 15 participants. We recruited 19 participants (14 females and five males; mean age, 20.1 years; range, 18–24 years). All participants were undergraduate psychology students at KU Leuven. They received course credits for their participation. Performance was higher than 95% correct in the subitizing trials for all participants. No data were removed from the analysis.

#### Apparatus and stimuli

**Figure 9.**
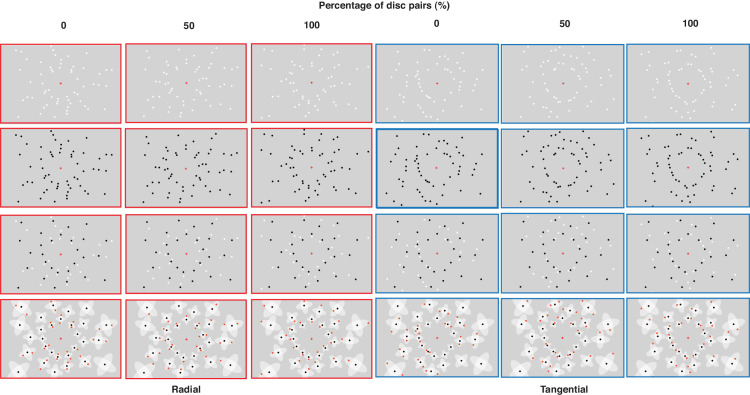
Illustration of displays in the radial and tangential conditions (first and third rows, respectively) and their geometric principles (second and last rows, respectively) in Experiment 4.

The apparatus was identical to that for Experiment 2. The stimuli in Experiment 4 included displays used in all the previous experiments, excluding the percentage of disc pairs of 25% and 75% (Figure 9). This was done to reduce the number of presented displays in each block (to limit the experiment duration to not exceed 120 minutes). The mixed contrast polarity displays used in Experiment 4 were identical to those in Experiment 3. Half of the uniform contrast polarity displays in Experiment 4 were identical to the displays in Experiment 1 (black discs); the other half consisted of white discs.

#### Design and procedure

The design and procedure were identical to those for Experiment 1 except for the following changes: (a) Participants viewed both small (34–44) and large (54–64) numerosities displays, as well as both uniform displays and mixed displays (within-subjects design); and (b) each participant completed eight blocks (four large numerosities blocks and four small numerosities blocks) of 144 trials in random order.

#### Data analysis

We performed a linear mixed-effects analysis on the obtained data. Arrangement, contrast polarity, and numerosity were submitted as fixed factors in the model. We also included the interaction between arrangement and contrast polarity as a fixed factor to test for an interaction between them. We did not include interaction terms involving numerosity to simplify the model and focus on the primary variables of interests. The model included both random slopes and random intercepts; that is, participants had their individual DV baselines, and the effects of arrangement, contrast polarity, and numerosity on DV varied across participants. Each factor was coded using simple contrast coding, with levels set to –0.5 (small numerosities, radial arrangement, and mixed contrast polarity) and 0.5 (large numerosities, tangential arrangement, and uniform contrast polarity). The coding scheme was chosen to enable comparisons between each variable's levels.

### Results

**Figure 10.**
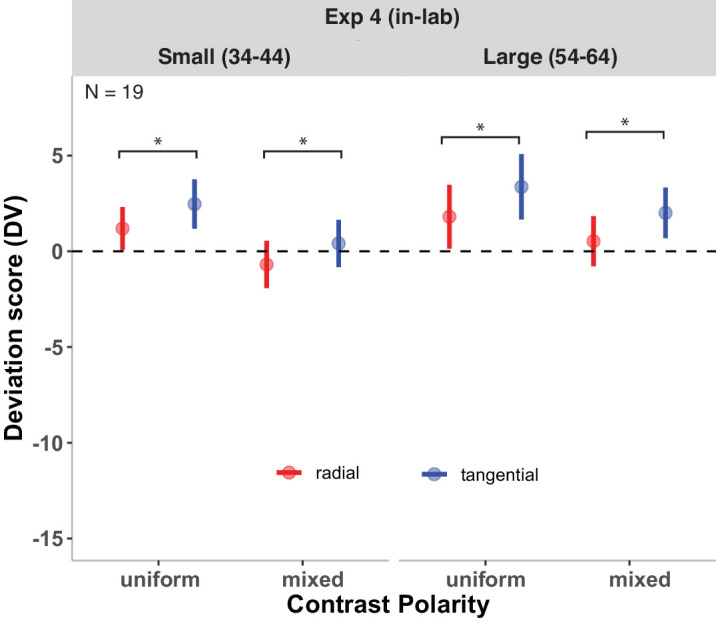
Results of Experiment 4. DVs of mixed and uniform displays in the radial and the tangential arrangements. DVs of 0 represent correct estimates; negative DVs, underestimations; and positive DVs, overestimations. Error bars indicate ±1 *SEM*. Significant fixed effects are denoted by *p < 0.05.

Figure 10 shows the results of Experiment 4. The analysis revealed a significant main effect of arrangement, *b* = 1.35 ± 0.33, *t*(81.15) = 4.16, *p* < 0.001, indicating that the DVs of radial arrangements were significantly lower compared with those for tangential arrangements (by on average 1.35), keeping other factors constant. There were no DV differences between displays that consisted of only white or only black discs. Hence, we averaged the results from both types of uniform contrast polarity displays. Contrast polarity exhibited a significant effect, *b* = 1.64 ± 0.61, *t*(19.00) = 2.68. *p* < 0.05, suggesting that DVs of mixed contrast polarity displays were lower compared with uniform contrast polarity displays. DVs of different numerosities did not significantly differ from each other, *b* = 1.08 ± 4.64, *t*(19.00) = 0.66, *p* = 0.52. There was no interaction between arrangement and contrast polarity, *t*(95.00) = 0.20, *p* = 0.84, indicating that the effect of contrast polarity on DVs was consistent regardless of whether displays were arranged radially or tangentially.

## Discussion

We investigated how local interaction between items modulated numerosity perception. For that aim, we created displays with varying numbers of items (ranging from 34 to 99 in the four experiments), predominantly arranged either radially or tangentially. In Experiment 1, the displays were composed of single discs, disc pairs, and disc triplets (Figure 2B) to vary the likelihood of local interactions among items. In Experiment 2, the displays consisted exclusively of disc triplets, maximizing the likelihood for local interference in the radial arrangements. In Experiment 3 and Experiment 4, the displays contained discs that were of opposite contrast polarity. In particular, flanking discs were of opposite contrast polarity (white) to the base discs (black). The mixed contrast polarity displays were expected to reduce local interference between neighboring discs and to reduce the perceived structural differences between radial and tangential displays. Other visual properties (convex hull, occupancy area, and density) of the displays were kept as similar as possible in the radial and the tangential displays in all experiments (Supplementary Table S1). In all four experiments, we asked participants to estimate the number of presented discs. We found that the estimates were lower when the discs were arranged radially compared with when they were arranged tangentially in all experiments. This result suggests that radial arrangements consistently yield lower numerosity estimates than tangential arrangements.

In Experiment 1, we expected higher interference among discs in the radial arrangements compared with the tangential arrangements, resulting in relative underestimations in the radial compared with the tangential arrangements. Our results were in line with this prediction: Estimates were lower for radial displays than for tangential displays, for both small (34–44 discs) and large (54–64 discs) numerosities. In Experiment 2, we investigated how numerosity estimation was impacted when using displays constructed to maximize the interference between the base discs and flanking discs. To maximize interference, there were always two flanking discs (instead of one or none) placed into the interference zones of the base discs. We observed results consistent with those of Experiment 1: Numerosity estimates were lower in the radial compared with the tangential arrangements in both numerosity ranges.

Comparing the results of Experiment 1 (weak radial and tangential arrangements) and Experiment 2 (strong radial and tangential arrangements) showed that estimates were similar in the two experiments, suggesting that maximizing the probability of local interference among discs did not modulate (decrease) numerosity estimates. Hence, our results showed that the radial–tangential anisotropy was also strong when the probability of local interference between discs was not maximized: Not every base disc had to be flanked by two discs (forming triplets, the expected strongest interference condition in our manipulation) for lower estimates in the radial than the tangential displays. When only one disc was placed into the interference zone (disc pairs), interference would be expected to be lower than for disc triplets. This expectation is based on findings in redundancy masking (RM) and crowding paradigms. In RM, interference occurs with three items but has not been shown with two items, suggesting lower (or no) interference with two items compared with three (Yildirim et al., 2021). Similarly, in crowding, if only one flanker is presented, crowding is weaker compared with two flankers (one on each side) (Huckauf & Heller, 2002; see also Manassi, Sayim, & Herzog, 2012). However, when large numbers of items are presented covering many locations in the visual field simultaneously, as was the case with our displays, interference that yields a reduction of the number of perceived items might occur with smaller numbers of neighboring items (such as disc pairs). Another explanation for this pattern of results could be that interference between radially arranged discs (which would manifest in low numerosity estimates) was already high in Experiment 1, approaching a plateau that could not be surpassed in Experiment 2. Alternatively, different grouping patterns (such as the more pronounced formations of ray and concentric patterns in Experiment 2) may also have modulated estimations (see also discussion below).

Experiment 3 was identical to Experiment 1, except that mixed contrast polarity displays instead of uniform contrast polarity displays were used. Mixed contrast polarity displays were expected to reduce the local interference between flanking and base discs and to break visual structures that emerge when items are perceived to be grouped (see Im, Zhong, & Halberda, 2016). Hence, mixed contrast polarity displays allowed us to investigate whether they produced less interference among items compared with uniform contrast polarity displays: If discs of opposite contrast polarity interfered less (or not at all) with neighboring discs, the difference between radial and tangential displays in regard to numerosity estimates would be expected to be lower or absent. In particular, this interaction between arrangement and contrast polarity was expected based on results in RM studies where RM was very weak for triplets with alternating contrast polarity compared with uniform contrast polarity (Hansmann-Roth & Sayim, 2023) and crowding studies which showed that flankers of opposite contrast polarity resulted in weaker crowding than flankers of the same contrast polarity as the target (Chakravarthi & Bertamini, 2020; Chung & Mansfield, 2009; Chung et al., 2001; Kooi et al., 1994; Sayim et al., 2008). Interestingly, the pattern of results in Experiment 3 was similar to that in Experiments 1 and 2 with lower estimates for radial than for tangential displays, showing that the radial–tangential anisotropy of numerosity perception persisted with mixed contrast polarity displays.

There are several potential explanations for this result. Critically, it has been shown that opposite contrast polarity does not result in improved performance in a crowding paradigm where participants reported all peripherally presented targets (“full report”) (Rummens & Sayim, 2021). Rummens and Sayim (2021) investigated whether the advantage in opposite contrast polarity conditions compared with uniform (same contrast polarity) conditions occurred when not a single (central) target had to be reported but all three items (the target and two flankers). The stimuli were either of uniform contrast polarity (e.g., black, black, black) or of mixed contrast polarity (e.g., black, white, black). Participants reported either the central item or the entire triplet. The results showed that performance in the mixed contrast polarity condition was better compared with the uniform condition when reporting the central item (the “opposite polarity advantage”). Importantly, when reporting all three items, performance was better in the uniform contrast polarity condition compared with the opposite contrast polarity condition. Hence, when all items were task relevant, the typical advantage with opposite contrast polarity ceased. The findings of our study are in line with these results: When estimating the number of items, all items are task relevant. Hence, task relevance may be an important factor determining whether or not the local spatial interactions of items are deleterious (but see discussion on RM below). Furthermore, research showed that, when the target was surrounded by multiple equidistant flankers that were alternating in contrast polarity, crowding was not reduced as with single flankers of opposite contrast polarity (Sayim et al., 2008). Sayim et al. (2008) suggested that multiple mixed contrast polarity flankers override the local dissimilarity (i.e., a target with neighboring opposite contrast polarity flankers) by grouping with the target and therefore result in strong crowding. Finally, recent results in a study on RM showed that, although RM was strongly reduced for triplets with alternating contrast polarity compared with uniform stimuli, it was similarly strong for alternating and uniform stimuli when more lines were presented (Hansmann-Roth & Sayim, 2023). Moreover, the number of lines in tangential displays in a RM paradigm tends to be overestimated (Yildirim et al., 2020). This effect could be similar or more pronounced with larger numbers of items as in the present study, thereby maintaining a significant difference between radial and tangential arrangements even when RM for radial arrangements of small numbers of items was reduced. To what extent mixed contrast polarity modulates RM in tangential displays is unclear; however, as RM seems not to occur in tangential arrangements (Yildirim et al., 2020), no reduction of RM is possible.

Another goal of the manipulation of contrast polarity was to increase the structural similarity of the two types of displays. In particular, opposite contrast polarity within displays was used to reduce the local grouping between the discs (discs pairs and triplets) (Pinna, Conti, & Porcheddu, 2021; Sayim et al., 2008; Spehar, 2002) and to break the structures of predominantly ray patterns in the radial arrangements and concentric patterns in the tangential arrangements. For example, Pinna et al. (2021) showed how opposite contrast polarity is a highly efficient cue for (un-)grouping, dominating many other grouping cues. Here, the use of opposite contrast polarity effectively disrupted the predominant ray patterns in the radial arrangements and concentric patterns in the tangential arrangements, as demonstrated in Figure 9: Radial and tangential displays with discs of opposite contrast polarity appear more similar than radial and tangential displays with discs of uniform contrast polarity. We found consistent results for mixed and uniform contrast polarity, with lower estimates in radial compared with tangential arrangements. This suggests that grouping into ray and concentric patterns does not underlie the radial–tangential anisotropy of numerosity estimation.

In Experiment 4, participants viewed similar displays as in Experiments 1 and 3 in a within-subjects design. Experiment 4 aimed to explore the impact of contrast polarity on numerosity estimation while controlling for individual differences between participants. We found that radial displays were consistently estimated as less numerous compared with tangential displays for small (34–44 discs) and large (54–64 discs) numerosities in both uniform and mixed contrast polarity displays (no interaction between arrangement and contrast polarity), replicating the pattern of results in Experiments 1 and 3. Furthermore, estimates for mixed contrast polarity displays were lower than those in uniform contrast polarity displays. The results are consistent with the previous findings by Chakravarthi and Bertamini (2020), who found that displays with mixed contrast polarity were perceived as less numerous than those with uniform contrast polarity (particularly when the density of displays was relatively low). However, the results, as for the results by Chakravarthi and Bertamini (2020), are not in line with the numerosity estimation findings by Tibber et al. (2012), who showed that estimations in mixed and uniform contrast polarity displays were comparable (see also Dakin et al., 2011). They argued that similar numerosity estimations with uniform and mixed contrast polarity displays could be attributed to the capacity of the visual system to normalize contrast variations when making numerosity (or density) estimations. Our results were not expected based on RM accounts (and common crowding accounts; but see Rummens & Sayim, 2021). First, estimates were expected to be larger with mixed contrast polarity because of reduced local spatial interactions. Second, regarding the interaction between arrangement and contrast polarity, radial arrangements would be affected more by mixed contrast polarity displays compared with uniform contrast polarity displays than tangential arrangements. More specifically, tangential arrangements were not expected to benefit from mixed contrast polarity compared with uniform displays because there is no RM in tangential displays. Common crowding accounts would predict either no or a weak interaction between arrangement and contrast polarity, depending on the magnitude of the reduction of crowding with mixed contrast polarity in the radial and tangential arrangements, respectively. The opposite pattern of results in full report paradigms in crowding (that is, better performance with uniform than mixed contrast polarity stimuli) (Rummens & Sayim, 2021; see also Chung & Mansfield, 2009; Rummens & Sayim, 2019; Rummens & Sayim, 2022) is in line with the main effect of contrast polarity in the present study: Worse performance with mixed than uniform contrast polarity in crowding would predict lower estimates (stronger interference) with mixed compared with uniform contrast polarity in numerosity perception. The prediction of an interaction between arrangement and contrast polarity, however, would again depend on the magnitude of crowding differences in uniform and mixed displays in the radial and tangential arrangements. Taken together, factors that do not significantly impact performance in RM and crowding studies seem to modulate numerosity estimates, and differences among typical RM, crowding, and numerosity estimation paradigms may well play a role here. Crowding paradigms generally require the identification (or discrimination) of a single flanked target (single report) or all presented items (full report) (e.g., Rummens & Sayim, 2021). In numerosity studies, by contrast, no identification or discrimination is necessary. As in RM paradigms, numerosity estimation requires only detection of the presented items. Differences in attentional allocation—typically concentrated on small areas in RM and crowding paradigms versus a broader focus in numerosity studies—may also influence performance in the different paradigms and are likely to reduce the predictive performance of findings in RM and crowding for numerosity studies.

Previous studies have shown that the number of items in a cluster tends to be underestimated (e.g., Chakravarthi & Bertamini, 2020; Frith & Frith, 1972; Ginsburg, 1980; Ginsburg & Goldstein, 1987). Yet, research has also shown overestimation (Alam, Luccio, & Vardabasso, 1986; L-Miao et al., 2022). In the current study, the overestimations and underestimations are inconsistent across the different experiments. Experiment 1 and Experiment 3 exhibited a trend toward underestimation, whereas Experiment 2 and Experiment 4 showed a trend to overestimate. Importantly, despite these differences, we consistently observed that the radial displays were estimated as less numerous compared with the tangential displays. One possible explanation is that, due to the direct estimation procedure, there was a central response tendency where responses tend to be biased toward the mean of the distribution (Brady & Alvarez, 2011; Hollingworth, 1910). Another explanation could be that estimations are based on a distribution that consists of both sensory estimates and a priori hypotheses about the stimuli, and, here, the reference displays at the beginning of each block acted as estimation priors, potentially calibrating the participants’ estimates (Anobile et al., 2019; Izard & Dehaene, 2008).

Beyond our main question regarding differences between radial and tangential arrangements, we also explored accuracy. In particular, our comparison between Experiments 1 and 2 showed that there were no significant accuracy differences between experiments. The comparisons between Experiments 1 and 3 showed that estimates in Experiment 1b (large numerosities) were more accurate than those in Experiments 1a, 3a, and 3b. More specifically, estimates in the tangential condition of Experiments 1b were the most accurate. Conversely, in Experiment 4, radial arrangements were estimated more accurately than tangential arrangements. It is important to note that, independent of the accuracy in any condition, the key question concerns differences between the radial and tangential arrangements: Whereas we expected relative underestimation of radial compared with tangential arrangements in uniform displays, we expected no (or a smaller) difference between the radial and tangential arrangements in mixed contrast polarity displays. Concluding that either radially or tangentially arranged items can be estimated more accurately based on the current results seems premature (see also discussion on reference displays above).

Taken together, we observed in all experiments that predominantly radially arranged discs were estimated to be less numerous compared with tangentially arranged discs, demonstrating a strong radial–tangential anisotropy of numerosity perception. The radial–tangential anisotropy was also observed with displays of mixed contrast polarity, which had been expected to reduce or abolish the difference between estimations of radial and tangential arrangements. Although the observed radial–tangential anisotropy in uniform displays is in line with what would be expected if crowding or RM modulated numerosity perception, the results with mixed contrast polarity are not readily explained by these mechanisms. One reason for this is that there are mixed results in crowding studies: The polarity advantage occurred mainly with single targets and irrelevant flankers, but even then only if the target did not group with the flankers (as in alternating patterns with multiple flankers) (Sayim et al., 2008). On the other hand, when all targets were task relevant, as in numerosity perception, there was no polarity advantage (Rummens & Sayim, 2021). Redundancy masking with stimuli consisting of only a few (three) items yielded a polarity advantage and hence would predict a weaker (or no) difference between radial and tangential displays. However, the polarity advantage ceased for larger numbers of items (Hansmann-Roth & Sayim, 2023). Finally, our results suggest that grouping of the displays in predominantly ray or concentric patterns does not underlie the observed difference between radial and tangential arrangements, neither in uniform displays (L-Miao et al., 2022) nor in displays with mixed contrast polarity (as shown here). The underlying mechanisms of the radial–tangential anisotropy of numerosity perception thus remain unclear. The difference in attentional allocation, which is typically focused on small, localized areas in crowding and RM paradigms and broader in numerosity studies, may well play a role and should be further investigated in future studies. We suggest that numerosity perception is subject to a strong radial–tangential anisotropy possibly due to local spatial interactions between neighboring items.

## Supplementary Material

Supplement 1
