# Supplementary material for: The radial–tangential anisotropy of numerosity perception
Source: J Vis. 2024 Jul 24;24(7):15. doi: 10.1167/jov.24.7.15 (PMC11271808; doi:10.1167/jov.24.7.15)
Supplement: Supplement 1 [file jovi-24-7-15_s001.pdf]

Supplemental Materials

Supplementary Table S1

A summary of physical properties for the radial and tangential displays across tested numerosity ranges.

	Experiments 1, 3 4 displays				Experiment 2 displays			
	Numerosity (34-44)		Numerosity (54-64)		Numerosity (51-72)		Numerosity (78-99)	
	Tan*(SD)*	Rad*(SD)*	Tan*(SD)*	Rad*(SD)*	Tan*(SD)*	Rad*(SD)*	Tan*(SD)*	Rad*(SD)*
Convex hull (°))	49.95*(2.35)*	50.66*(2.57)*	76.29*(2.43)*	77.33*(2.94)*	51.77 *(1.84)*	52.95*(2.11)*	78.27*(2.73)*	79.74*(2.17)*
Occupancy area(Convex hull 2D volume)	170.38*(15.03)*	170.46*(15.04)*	400.72*(23.33)*	400.20*(23.51)*	186.16*(12.06)*	186.28*(12.70)*	431.60*(19.01)*	430.34*(25.81)*
Density (item/deg^2)	0.27*(<0.01)*	0.27*(<0.01)*	0.16*(<0.01)*	0.16*(<0.01)*	0.38*(0.02)*	0.38*(0.02)*	0.22*(0.01)*	0.22*(0.01)*

Note. Tan: Tangential displays; Rad: Radial displays. SD: Standard deviation. Convex hull and occupancy area were computed using the Qhull library (Barber et al., 1996) with Python. Density was calculated using the numerosity divided by occupancy area, excluding the central zone (24.3 deg^2) where no disc was presented
